# MtDNA meta-analysis reveals both phenotype specificity and allele heterogeneity: a model for differential association

**DOI:** 10.1038/srep43449

**Published:** 2017-02-23

**Authors:** Shani Marom, Michael Friger, Dan Mishmar

**Affiliations:** 1 Department of Life Sciences, Faculty of Natural Sciences, Ben-Gurion University of the Negev, Beer-Sheva, 8410501, Israel; 2 Department of Public Health, Faculty of Health Sciences, Ben-Gurion University of the Negev, Beer-Sheva, 8410501, Israel

## Abstract

Human mtDNA genetic variants have traditionally been considered markers for ancient population migrations. However, during the past three decades, these variants have been associated with altered susceptibility to various phenotypes, thus supporting their importance for human health. Nevertheless, mtDNA disease association has frequently been supported only in certain populations, due either to population stratification or differential epistatic compensations among populations. To partially overcome these obstacles, we performed meta-analysis of the multiple mtDNA association studies conducted until 2016, encompassing 53,975 patients and 63,323 controls. Our findings support the association of mtDNA haplogroups and recurrent variants with specific phenotypes such as Parkinson’s disease, type 2 diabetes, longevity, and breast cancer. Strikingly, our assessment of mtDNA variants’ involvement with multiple phenotypes revealed significant impact for Caucasian haplogroups H, J, and K. Therefore, ancient mtDNA variants could be divided into those that affect specific phenotypes, versus others with a general impact on phenotype combinations. We suggest that the mtDNA could serve as a model for phenotype specificity versus allele heterogeneity.

The mitochondria are the main source of energy production via the oxidative phosphorylation system (OXPHOS) and play a major role in cell life and death^1^, the differentiation of most energy-consuming tissues, and even the emergence of new species^2^. The mitochondrion is the only organelle in animal cells that harbors its own genome (mtDNA), which is likely an ancient remnant of its alpha-proteobacterial ancestor^3^,^4^. Mitochondrial DNA is present in multiple copies per cell that vary in number between individuals and tissues^1^. Human mtDNA is 16,569 bp in length, and consists mainly (93% in humans) of genes that encode for 13 protein subunits of the OXPHOS complexes (I, III–V), two ribosomal RNAs (*12SrRNA* and 1*6SrRNA*), and 22 tRNAs. The gene content of vertebrate mtDNA has remained stable throughout evolution, although gene order may vary^5^. Known gene regulatory elements are found mainly within the non-coding portion of the mtDNA (∼7% in humans), although recent findings imply that some mtDNA gene coding sequences have dual roles; they both code for genes and are bound by transcription factors and may thus have potential regulatory functions^6^.

The small number of genes encoded by the mtDNA is not sufficient to drive mitochondrial activities. Indeed, most genes (*N* = ~1500) required for mitochondrial function (such as apoptosis, nucleotide biosynthesis, fatty acid metabolism, iron metabolism, and so on) and for regulation of mtDNA transcription and replication are encoded by the nuclear genome (nDNA)^7^,^8^. These genes are translated in the cytoplasm, and in turn are imported into the mitochondrion^7^,^9^. Due to the bi-genomic origin of mitochondrial components, mutations in both mtDNA- and nDNA-encoded genes may lead to inherited metabolic disorders and increased susceptibility to complex diseases^10^. Thus, maintaining the efficient interaction between the factors encoded by the two genomes (mito-nuclear interactions) contribute to human health^11^. and in some cases may promote healthy, successful longevity^12^.

The association of mtDNA genetic variants with altered disease susceptibility has been reported in a wide range of clinical phenotype, most prominently Alzheimer’s disease (AD)^13^, Parkinson’s disease (PD)^14^, Type 2 diabetes mellitus (T2DM)^15^, Age-related macular degeneration (AMD)^16^, schizophrenia^17^, breast cancer, prostate cancer, pancreatic cancer^18^, multiple sclerosis (MS)^19^, myocardial infarction (MI)^20^, radiographic progression of knee osteoarthritis^21^, sperm motility^22^ and coronary artery disease (CAD)^23^, but also with non-clinical phenotypes such as longevity^24^ and the enhanced capabilities of elite athletes^25^ (Table 1) (and see also, references in Supporting Information Tables S2). Thus, mtDNA variants appear to be associated with a wide variety of phenotypes, in consistence with a major pleiotropic impact of mitochondrial variation on human health. Nevertheless, there are inconsistencies in the reported association of mtDNA genetic backgrounds (haplogroups) with phenotypes such as longevity^26^, AD^27^, T2DM^28^, breast cancer^29^, and prostate cancer^30^. This phenomenon interferes with our ability to understand the functional impact of mtDNA variants that stems from either epistatic interactions or population stratification. Such inconsistencies could be resolved, at least in part, by conducting large-scale meta-analysis.

Here, we aim to partially overcome these inconsistencies via meta-analysis of a large collection of publicly available data from association studies. We employed stringent quality control to assess the role of common human mtDNA variants in the genetic basis of a variety of phenotypes. The results emphasize allelic heterogeneity in the human mitochondrial genome – whereas some haplogroups associate with specific phenotypes, others have a pleiotropic impact.

## Results

### Association of mtDNA haplogroups with neurodegenerative diseases

**Figure 1:**
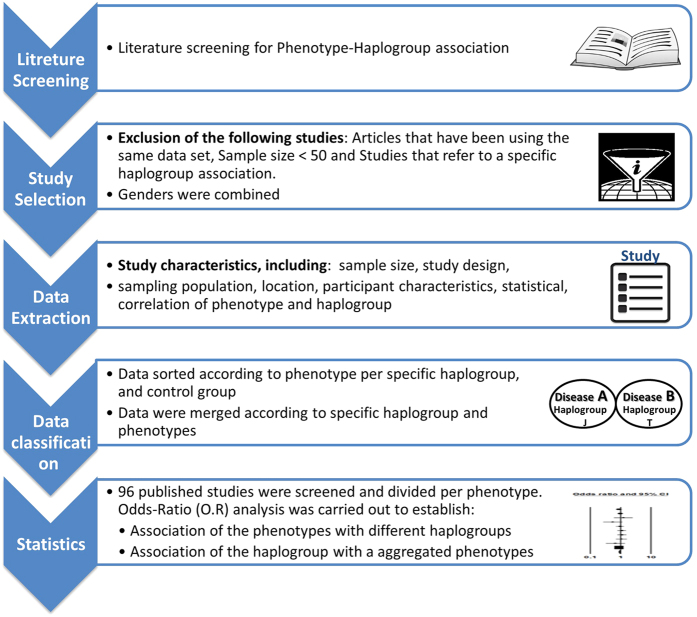
Work flow of our meta-analysis.

As the first step of our meta-analysis, we employed stringent quality-control criteria as we sifted through published studies (Fig. 1). Thus, we were able to focus our subsequent analysis on a specific subset of reported mtDNA-phenotype associations. Notably, most studies focused on populations with Caucasian ancestry.

**Figure 2:**
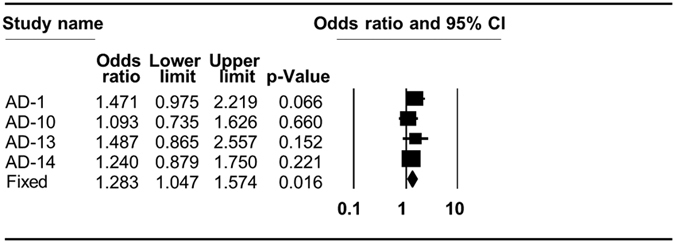
Forest plot of the association of mtDNA haplogroups with AD in Caucasians. AD–Alzheimer’s disease. The overall (fixed) significant P-values for the calculated odds ratios are framed. Shown are the results of haplogroup H analysis (see also Table 2).

**Figure 3:**
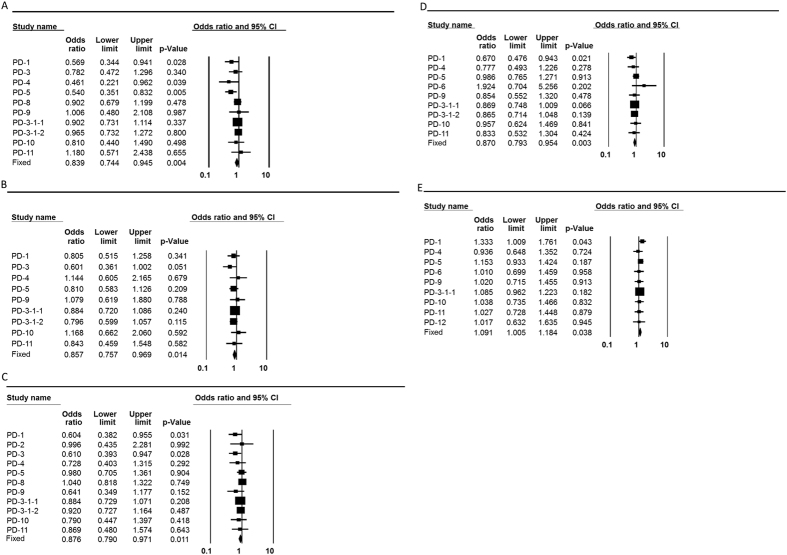
Forest plots for the association of mtDNA haplogroups with PD in Caucasians. PD–Parkinson’s disease. The overall (fixed) significant P-values for the calculated odds ratios are framed. (**A**) Haplogroup K analysis. (**B**) Haplogroup T analysis. (**C**) Haplogroup J analysis. (**D**) Haplogroup cluster JT analysis. (**E**) Haplogroup cluster HV analysis.

After correcting for heterogeneity among the studies (see Methods), we found that only Caucasian individuals with haplogroup H had a significantly increased risk of developing AD (pooled OR = 1.283, p = 0.016, 95% CI (1.047–1.574)) (Fig. 2, Table 2, and Supplementary Figure S1). While considering PD, we found that individuals with mtDNA haplogroup K had a significantly reduced risk of developing PD (pooled OR = 0.839, p = 0.004, 95% CI (0.744–0.945)), as did individuals with either mtDNA haplogroup T (pooled OR = 0.857, p = 0.014, 95% CI (0.757–0.969)), or haplogroup J (pooled OR = 0.876, p = 0.011, 95% CI (0.79–0.971)) (Table 2 and Fig. 3). Notably, grouping haplogroups J and T due to phylogenetic considerations increased the strength of the reduced risk for PD, but did not change the effect size (pooled OR = 0.87, p = 0.003, 95% CI (0.793–0.954)) (Table 2 and Fig. 3). A significantly increased risk of developing PD was identified for individuals who belong to haplogroup cluster HV (pooled OR = 1.091, p = 0.038, 95% CI (1.005–1.184)) (Table 2 and Fig. 3). These results are precisely in line with previous meta-analysis performed for PD, and thus validate our approach^31^.

### Mitochondrial DNA haplogroups are associated with successful longevity

**Figure 4:**
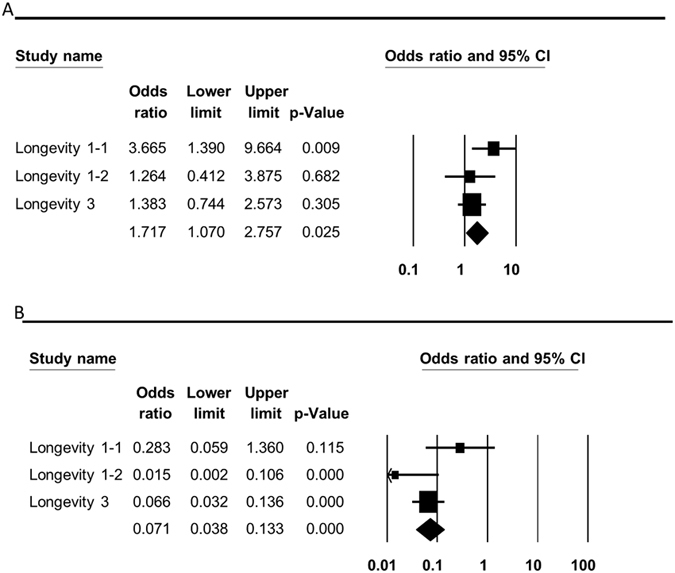
Forest plots for the association of mtDNA haplogroups with longevity in Caucasians. The overall (fixed) significant P-values for the calculated odds ratios are framed. (**A**) Haplogroup J analysis. (**B**) Haplogroup W analysis.

Our analysis confirmed the significant association of haplogroup J individuals with successful longevity (pooled OR = 1.717, p = 0.025, 95% CI (1.07–2.757)), as the frequency of haplogroup J was notably higher in centenarians as compared to younger individuals in various Caucasian populations. In contrast, haplogroup W individuals had significantly reduced odds of aging successfully (OR = 0.071, p = 0.000, 95% CI (0.038–0.133)) (Table 2, Fig. 4 and Supplementary Figure S2). Our analysis of Asian studies did not confirm any significant association between longevity and mtDNA haplogroups (Table 2 and Supplementary Fig. S2).

### A worldwide recurrent common mtDNA variant, as well as mtDNA haplogroups in Asians, alter susceptibility to developing T2DM and breast cancer

**Figure 5:**
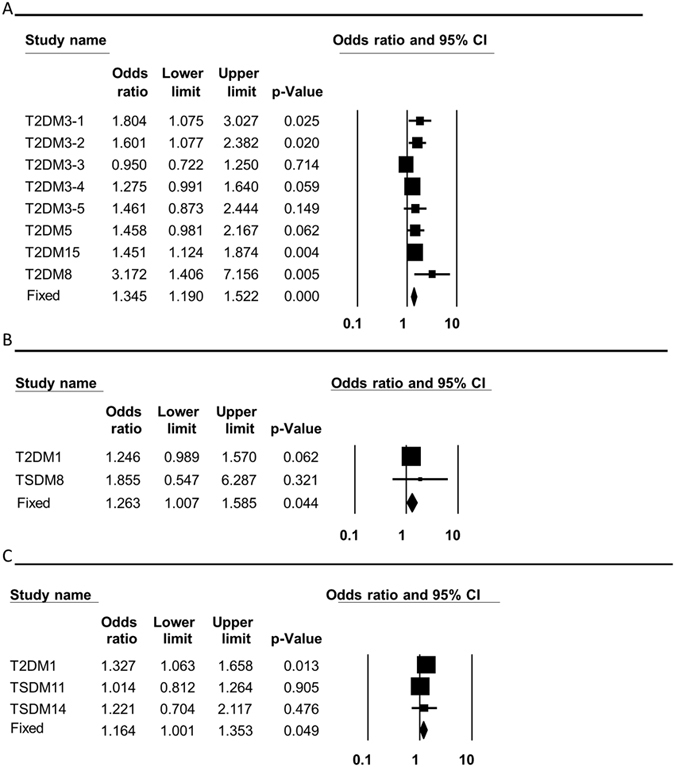
Forest plots for the association of mtDNA haplogroups with T2DM in Asians. T2DM–type 2 diabetes mellitus. The overall (fixed) significant P-values for the calculated odds ratios are framed. (**A**) Variant 16,189C analysis. (**B**) Haplogroup A analysis. (**C**) Haplogroup F analysis.

**Figure 6:**
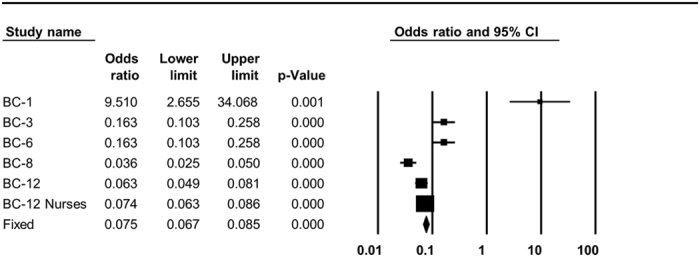
Forest plots of the association of mtDNA haplogroups with breast cancer in Caucasians. The overall (fixed) significant P-values for the calculated odds ratios are framed. Shown is the analysis of 10,398G variant.

Our analysis of studies performed in Caucasians as a whole did not support any association between T2DM and any mtDNA haplogroup (Table 2 and Supplementary Figure S3). However, when we analyzed the data from studies that looked for correlation of mtDNA haplogroups with T2DM in Asian populations, we identified an increased risk of developing T2DM in haplogroups A (pooled OR = 1.263, p = 0.044, 95% CI (1.007–1.585)) and F (pooled OR = 1.164, p = 0.049, 95% CI (1.001–1.353)) (Table 2 and Fig. 5). In addition, we confirm that the common recurrent variant 16,189C is associated with an increased risk of T2DM (pooled OR = 1.345, p = 0.000, 95% CI (1.190–1.522)) (Table 2 and Fig. 5)^32^. For haplogroup D5 and G, no association with T2DM could be supported (Supplementary Figure S4). In addition, our meta-analysis reported significant reduction in breast cancer risk for individuals who carry the 10,398G variant (pooled OR = 0.075, p = 0.000, 95% CI (0.067–0.085)) (Table 2 and Fig. 6).

### Certain mtDNA haplogroups are associated with multiple phenotypes, suggesting pleiotropism

**Figure 7:**
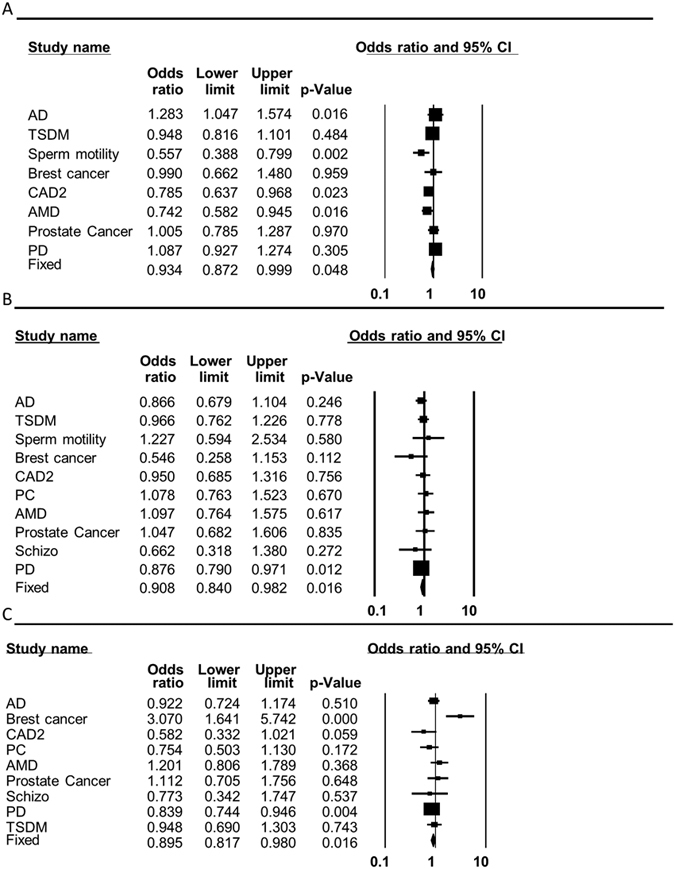
MtDNA haplogroup association with grouped phenotypes. Forest plots for the analysis of the different phenotypes are shown. The overall (fixed) significant P-values for the calculated odds ratios are framed. (**A**) Haplogroup H analysis. (**B**) Haplogroup J analysis. (**C**) Haplogroup K analysis.

The risk of developing most common complex human diseases is influenced by multiple genetic and environmental factors, which are frequently shared between different phenotypes. It is thus very possible that a given SNP will be associated with more than a single phenotype. A close inspection of our results revealed that certain haplogroups had a significant impact on a repertoire of phenotypes, consistent with pleiotropism. While testing for the global impact of mtDNA genetic background on diverse phenotypes, we found significant global phenotypic impact of haplogroup H (pooled OR = 0.934, p = 0.048, 95% CI (0.872–0.999)), haplogroup K (pooled OR = 0.895, p = 0.016, 95% CI (0.817–0.98)), and haplogroup J (pooled OR = 0.908, p = 0.016, 95% CI (0.84–0.982)) (Table 3 and Fig. 7). No such global association was identified for the other haplogroups (Table 3 and Supplementary Figure S5). This result strongly suggests that the mutations that define these three Caucasian mtDNA haplogroups have a wide functional impact, in stark contrast to mutations that define other haplogroups (such as haplogroup W), whose functional implications are apparently phenotype-specific.

## Discussion

**Figure 8:**
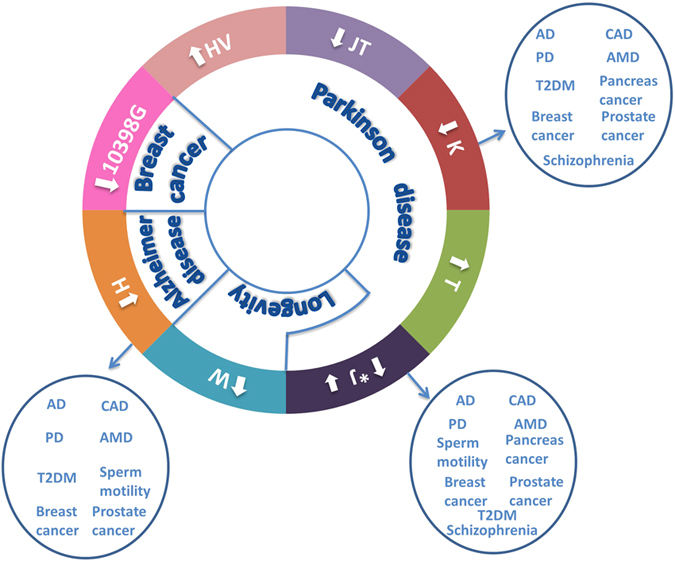
The phenotypic impact of mtDNA haplogroups–phenotype specificity versus pleiotropism. Arrows within the circles: Outward arrows–increased risk; inward arrows–reduced risk; *haplogroup J confers reduced risk to PD and supports successful longevity. Arrows outside the circle: altered susceptibility to an aggregate of phenotypes. The latter is observed in haplogroups H, J and K.

During the past several decades, many common genetic variants have been associated with altered susceptibility to develop complex diseases, yet occasionally diverse phenotypes share association with the same variant, suggesting pleiotropism^33^,^34^,^35^,^36^. Pleiotropism underlines the diffuse boundaries between phenotypes in general and diseases in particular^37^,^38^,^39^. This is especially true for complex disorders that share phenotypic characteristics, and hence may share some of the same molecular mechanisms^40^. Genetic variation in the human mitochondrial genome constitutes an extreme case of pleiotropism, and underlines its involvement in a diverse repertoire of phenotypes. Specifically, our meta-analysis demonstrated the strength of association of mtDNA haplogroups with complex disorders including PD, T2DM, AD, and breast cancer, in addition to successful longevity. Furthermore, our analysis demonstrated that unlike other haplogroups, the phenotypic impact of haplogroups H, J, and K remained significant even while grouping the disease phenotypes tested (Fig. 8). This finding is consistent with a pleiotropic effect of these three haplogroups. Alternatively, the latter result may only reflect the strong functionality of the mutations defining haplogroups H, J and K, which remained significant even after analyzing an aggregate of the phenotypes tested. Taken together, ancient mtDNA variants have clear impact on human health. Our results underline the important role of this maternally inherited genome in the underlying mechanisms of multiple complex diseases.

The underlying mechanism of the pleiotropic impact of variants is frequently ignored, mainly due to its apparent complexity and the resulting difficulty of investigating its molecular basis. This difficulty could be addressed, in part, by focusing on certain biochemical pathways. Mitochondrial-DNA-encoded factors constitute a good example of such pathways, as they are either protein members of the OXPHOS or RNA components of the mitochondrial translation system. Moreover, in contrast to the nuclear genome, mtDNA-encoded genes harbor many more variants due to the one order of magnitude higher mutation rate. Many of these variants have experimentally proven functional impact^41^,^42^,^43^,^44^,^45^, and support association with multiple phenotypes. Therefore, further assessment of the functional impact of mtDNA variants may serve as the first step towards understanding the molecular basis of pleiotropism.

Our meta-analysis not only strengthened the association of mtDNA variants with certain phenotypes, but also underlined their pleiotropic impact (Fig. 8). Specifically, whereas our phenotype-specific analysis underlined the association of mtDNA haplogroup K with PD, pooled odds ratio calculation (for all the phenotypic data) revealed that haplogroup K had an overall strong phenotypic impact, thus reflecting pleiotropism. Similar results were revealed for haplogroups J and H. Furthermore, close inspection of our data revealed that while haplogroup J is associated with protection against PD and support for successful aging, haplogroup H increases the susceptibility to certain age-related disorders (Table 2 and Fig. 8). These findings suggest a putative contrasting phenotypic impact of the mutations that define haplogroups J and H. We believe that future investigation of pleiotropism versus phenotype specificity of mtDNA variants (such as that of haplogroup W) may shed new light on the molecular impact of mtDNA mutations.

While they are encouraging, we stress that, like other meta-analyses, our findings may be affected by publication bias, mainly the traditional lack of publically available negative results. We were fortunate to be able to include such negative results in our analysis, especially regarding longevity^26^,^46^, AD^27^,^47^ (and see also, refs 1,10,13 and 14 in the Supporting Information) and prostate cancer^30^. A second source of publication bias may stem from over-representation of studies on association to PD, AD, and T2DM. We attempted to correct for this bias by calculating pooled odds ratios. A third possible problem may stem from mtDNA mis-annotation during haplogroup assignment in the studies included in our meta-analysis. Since we employed strict quality control criteria for data inclusion, which was based in part on haplogroup assignment methods, mis-annotation is expected to affect only a small proportion of the studies included in our analysis. Thus, we believe that such bias had only negligible impact on the current study. The only bias that we could not address was the overrepresentation of papers that focus on Caucasians, the relatively small representation of Asians, and the virtual absence of Africans. As the African population is very diverse, especially in terms of mitochondrial genomic sequences, more mitochondrial-based disease association studies from this continent will provide better insight on the contribution of mitochondrial genetic variation to disease.

Previous analysis in our lab demonstrated the functionality and possibly adaptive impact of recurrent ancient mtDNA variants during human evolution^48^. Some of these ancient recurrent variants (such as T3394C) not only had an adaptive potential in ancient times, but also altered susceptibility to human diseases today. Our current analysis further validated previous reports^15^,^49^ regarding the association of the highly recurrent variant T16,189C with susceptibility to T2DM in both Caucasians and Asians (Table 2 and Fig. 5). Since the T16,189C mutation likely appeared multiple times during human phylogeny, its adaptive role cannot be easily estimated.

Meta-analysis of mtDNA variants is blind to a major unique feature of mtDNA genetic variability–the distribution of mtDNA haplogroups may vary among closely related ethnic groups^50^. For example, association of mtDNA variants with complications of T2DM was identified only in certain Jewish populations^51^, and longevity was associated with haplogroup J only in certain Italian populations^24^. Therefore, while our meta-analysis overlooked population-specific effects of genetic variants, which in our case are considered false-negative results, it provided the ability to fortify the overall functionality of several common mtDNA variants. Our ability to support previous studies stems from the availability of a large data set. Hence, the apparent absence of significant association of mtDNA variants with more phenotypes does not necessarily indicate a lack of association, but rather underlines the need for additional data.

One of our main findings is that certain mtDNA variants associate with specific phenotypes, yet other mtDNA variants have a more general phenotypic impact. This reflects allelic heterogeneity in the mtDNA. Allelic heterogeneity has been demonstrated in several nuclear DNA loci, and could be divided into two subtypes: (A) *One gene, one mutation, many phenotypes*: For example, the C376T mutation within the *LGR4* (Leucine-rich repeat-containing G-protein coupled receptor 4 gene) is strongly associated with low bone mineral density (BMD), osteoporotic fractures, as well as with an increased risk to develop squamous skin cell carcinoma and biliary tract cancer^52^. (B) *One gene, many mutations, many phenotypes*: For example, different mutations in the *RET* gene have been implicated in the etiology of Hirshprung disease as well as with Type 2 Multiple Endocrine Neoplasia (MEN)^53^. Similarly, loss of function mutations within the *FGFR1* (fibroblast growth factor receptor 1) locus underlie the autosomal dominant form of Kallman syndrome, while gain of function mutations at the same site lead to a subtype of craniosynostosis (Pfeiffer syndrome)^54^,^55^. Indeed, our meta-analysis strongly supported both subtypes of mtDNA allelic heterogeneity. For example, the different variants defining haplogroups T and HV independently alter the susceptibility to Parkinson’s disease (Figs 3 and 8). Secondly, haplogroup J whose association was supported by our meta-analysis for a variety of phenotypes (Figs 7 and 8) is defined by variants of which some have experimentally-proven impact on mtDNA functional regulation^45^. Hence, our work, underline the mtDNA as an attractive model for allelic heterogeneity.

In summary, our analysis not only validated the association of mtDNA haplogroups with altered susceptibility to a variety of diseases and involvement with certain phenotypes, but also clearly underlined the pleiotropic impact of common mtDNA variants. Thus, it revealed that mtDNA variability stands out as a major player in allelic heterogeneity in humans.

## Materials and Methods

### Data sources and literature search

PubMed and Google Scholar search engines were used to identify publications (publically available until February 30, 2016) that describe association of mtDNA variants with phenotypes in humans. Supplementary Table S1 lists the details of our search strategies. No language restrictions were applied. All articles were reviewed to identify those that studied association between any phenotype and both mtDNA genetic backgrounds (haplogroup) and certain common variants. Our workflow is described in Fig. 1 and the full list of papers used divided according to the phenotypes are listed in Supplementary Tables S2–S11.

### Quality control criteria

To avoid bias, studies that addressed these relationships while focusing on a certain haplogroup from the start while grouping other genetic backgrounds as ‘others’ were excluded from further analysis. We also excluded articles whose analysis relied on the same datasets. Studies with sample sizes below N = 50 were excluded. We classified the phenotype-haplogroup association analysis according to major global population assignment (i.e., Caucasian, African, and Asian). Occasionally, depending on sample size available, we used more focused populations (such as Iranians). Since only a small subset of the studies analyzed samples while considering gender, to avoid sample size issues we included data regardless of gender division and considered samples while combining the two sexes. To avoid sample size issues we considered the phenotypes ‘Longevity’ and ‘Extreme Longevity’ as a single combined group. To reduce mis-annotation bias we used association studies in which haplogroup assignment was based on the combination of coding region SNPs and control region sequences. Notably, our above-mentioned quality control criteria led to exclusion of many association studies using mitochondrial phenotypes such as the enhanced capabilities of elite athletes and sperm motility.

### Data Extraction

Data was extracted while taking into account several study characteristics, including sample size, study design, sampling population, geographic location, participant characteristics (age and sex), statistical adjustment used, correlation of phenotype, and haplogroup. The data was sorted according to phenotypes per specific haplogroup (see examples in Supplementary Table S12). After classification of the sample data from the articles according to mtDNA haplogroups, we merged the data from studies separately according to phenotypes (Supplementary Table S13).

### Meta-analysis statistics

Meta-analysis was conducted using Comprehensive Meta-Analysis software, Version 3 (www.meta-analysis.com). In total, 96 published studies that included 53,975 patient and 63,323 controls were screened and divided according to phenotypes (Table 1). From each paper, sample information (i.e., sample sizes, cases vs. controls, and so on) was extracted per phenotype-haplogroup association study (Table 2 and Table 4). Calculation of odds ratios (OR) was carried out to establish the phenotype association with mtDNA haplogroups. Heterogeneity analysis was performed to assess differences or similarities between the included studies. Prior to estimating the combined effect that emerged from a group of studies, we checked for similarity between the identified effects in each of the individual studies, so that the combined estimate would reflect a meaningful description of the overall collection of studies per phenotype. To employ the heterogeneity statistic, the weighted sum of squares of the residuals was used as a generalization of Cochran’s Q from meta-analysis to meta-regression. Following this calculation, a test of the null hypothesis (i.e., no residual heterogeneity) was performed by comparing Cochran’s Q to a chi square distribution^56^,^57^. Heterogenic data was excluded from further analysis. OR was calculated and estimated for a significant pool of haplogroup-phenotype associations. OR was significant when p < 0.05.

## Additional Information

**How to cite this article**: Marom, S. *et al*. MtDNA meta-analysis reveals both phenotype specificity and allele heterogeneity: a model for differential association. *Sci. Rep.* **7**, 43449; doi: 10.1038/srep43449 (2017).

**Publisher's note:** Springer Nature remains neutral with regard to jurisdictional claims in published maps and institutional affiliations.

## Supplementary Material

Supplementary Information

## Figures and Tables

**Table 1:** Number of analyzed studies and sample sizes.

Phenotype	Number of studies	Sample size	
		Patients	Controls
AD	14	4180	3717
PD	13	9243	17999
T2DM	15	11031	9804
Longevity	15	3502	5123
Breast Cancer	14	11788	13192
Prostate Cancer	6	1393	1397
Sperm Motility	4	3292	180
Schizophrenia	2	295	986
AMD	4	778	3626
MS	4	3896	1255
MI	3	2367	1678
CAD	2	969	3008
PC	2	1241	1358
Total	**96**	**53975**	**63323**

**Table 2:** Meta-analysis of mtDNA haplogroup association with diverse phenotypes.

Phenotype	Haplogroup	OR	p-value
AD	H	1.283	**0.016**
	T	1.002	0.989
	J	0.987	0.92
	K	0.956	0.734
	U	1.018	0.85
	V	0.989	0.952
	W	0.992	0.975
	X	1.39	0.165
PD	K	0.839	**0.004**
	T	0.857	**0.014**
	J	0.876	**0.011**
	HV	1.091	**0.038**
	JT	0.87	**0.003**
T2DM	H	0.966	0.558
	U	1.074	0.377
	T	0.949	0.61
	J	1.078	0.742
	X	1.077	0.744
T2DM - Asian	16189C	1.345	**0.000**
	A	1.263	**0.044**
	D5	1.208	0.086
	F	1.164	**0.049**
	G	0.949	0.585
Longevity	H	0.843	0.141
	I	1.493	0.264
	J	1.717	**0.025**
	K	1.119	0.64
	T	0.882	0.575
	U	0.781	0.281
	W	0.071	**0.000**
	X	1.315	0.477
Longevity Asian	D	1.252	0.165
Breast Cancer	10398G	0.075	**0.000**

**Table 3:** MtDNA haplogroup association with grouped phenotypes.

Haplogroup	OR	p-Value
H	**0.934**	**0.048**
K	**0.895**	**0.003**
J	**0.908**	**0.016**
**I**	0.924	0.551
**T**	0.922	0.07
**U**	1.050	0.331
**V**	1.034	0.749
**W**	1.027	0.836
**X**	1.141	0.327

The significantly associated haplogroups are bolded.

**Table 4:** Available sample size per phenotype-mtDNA haplogroup association study.

Phenotype	Haplogroup	Patients per haplogroup (all patients)	Controls	Number of studies
AD	H	88 (185)	705 (2083)	6
	T	124 (1427)	151 (1650)	6
	J	126 (1427)	166 (1650)	6
	K	134 (1752)	152 (1844)	5
	U	287 (2363)	295 (2426)	7
	V	64 (1675)	56 (1457)	4
	W	38 (1935)	32 (1700)	5
	X	47 (1935)	30 (700)	5
T2DM	H	1031 (2484)	996 (2536)	4
	U	434 (2484)	349 (2536)	4
	T	210 (2484)	223 (2536)	4
	J	198 (2115)	177 (1850)	3
	X	45 (2115)	37 (1850)	3
T2DM (Asians)	16189C	1115 (3187)	346 (2126)	8
	A	207 (2952)	220 (3451)	3
	DS	161 (3115)	181 (3645)	3
	F	386 (3115)	412 (3645)	3
	G	226 (2952)	268 (3451)	3
Longevity	H	26 (502)	373 (813)	4
	I	16 (437)	17 (675)	3
	J	44 (502)	53 (813)	4
	K	32 (437)	45 (675)	3
	T	35 (502)	61 (813)	3
	U	99 (502)	150 (813)	3
	V	14 (502)	29 (813)	4
	W	11 (437)	15 (675)	3
	X	13 (437)	15 (675)	3
Longevity Asian	D	102(525)	184 (781)	3
Breast Cancer	10398G	656 (359)	791 (3751)	6
