# Supplementary material for: MtDNA meta-analysis reveals both phenotype specificity and allele heterogeneity: a model for differential association
Source: Sci Rep. 2017 Feb 23;7:43449. doi: 10.1038/srep43449 (PMC5322532; doi:10.1038/srep43449)
Supplement: Supplementary Information [file srep43449-s1.pdf]

Supplementary Information of:

MtDNA meta-analysis reveals both phenotype specificity and allele heterogeneity: a model for differential association

Shani Martsiano[1], Michael Friger[2], and Dan Mishmar[1*]

[1] Department of Life Sciences, Faculty of Natural Sciences, Ben-Gurion University of the Negev, Beer-Sheva, Israel 8410501

[2] Department of Public Health, Faculty of Health Sciences, Ben-Gurion University of the Negev, Beer-Sheva, Israel 8410501

*Corresponding author

Dan Mishmar, PhD

Department of Life Sciences

Ben-Gurion University of the Negev

Beer-Sheva

Israel 8410501

Tel: +972-8-6461355

FAX: +972-8-6461356

Email: dmishmar@bgu.ac.il

A **AD - Haplogroup T**

Study name	Odds ratio	Lower limit	Upper limit	p-Value
AD-1	1.379	0.767	2.482	0.283
AD-2	1.189	0.679	2.081	0.545
AD-9	0.977	0.523	1.826	0.942
AD-10	0.661	0.328	1.332	0.247
AD-13	0.658	0.230	1.881	0.435
AD-14	0.938	0.512	1.717	0.834
Fixed	1.002	0.769	1.305	0.989

Odds ratio and 95% CI

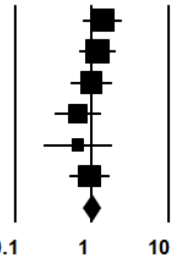

B **AD - Haplogroup J**

Study name	Odds ratio	Lower limit	Upper limit	p-Value
AD-1	1.088	0.525	2.254	0.821
AD-2	1.248	0.762	2.044	0.380
AD-9	0.781	0.439	1.389	0.400
AD-10	1.119	0.536	2.337	0.764
AD-13	2.806	0.570	13.814	0.205
AD-14	0.692	0.391	1.226	0.207
Fixed	0.987	0.757	1.285	0.920

Odds ratio and 95% CI

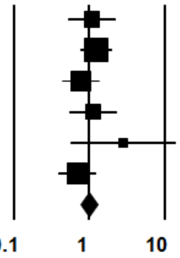

C **AD - Haplogroup K**

Study name	Odds ratio	Lower limit	Upper limit	p-Value
AD-1	0.569	0.264	1.226	0.150
AD-5	0.803	0.565	1.141	0.221
AD-9	0.966	0.551	1.691	0.902
AD-13	1.301	0.303	5.579	0.723
AD-14	1.419	0.806	2.497	0.225
Fixed	0.906	0.709	1.159	0.433

Odds ratio and 95% CI

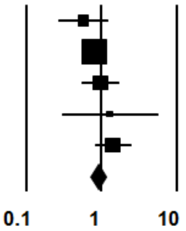

D AD - Haplogroup U

Study name	Odds ratio	Lower limit	Upper limit	p-Value
AD-1	1.010	0.538	1.895	0.976
AD-2	0.919	0.641	1.317	0.645
AD-5	0.984	0.609	1.590	0.947
AD-9	1.108	0.683	1.798	0.679
AD-10	1.121	0.627	2.003	0.700
AD-13	0.901	0.516	1.572	0.713
AD-14	1.173	0.748	1.838	0.487
Fixed	1.018	0.848	1.222	0.850

Odds ratio and 95% CI

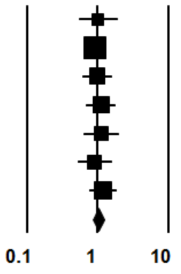

E AD - Haplogroup V

Study name	Odds ratio	Lower limit	Upper limit	p-Value
AD-2	1.031	0.524	2.031	0.929
AD-5	0.882	0.527	1.477	0.634
AD-10	2.104	0.637	6.947	0.222
AD-13	0.762	0.238	2.440	0.647
Fixed	0.989	0.684	1.429	0.952

Odds ratio and 95% CI

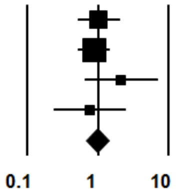

F AD - Haplogroup W

Study name	Odds ratio	Lower limit	Upper limit	p-Value
AD-2	1.300	0.289	5.846	0.732
AD-5	1.037	0.534	2.015	0.914
AD-9	3.781	0.420	34.069	0.236
AD-10	1.146	0.303	4.331	0.841
AD-13	0.462	0.146	1.460	0.189
Fixed	0.992	0.610	1.613	0.975

Odds ratio and 95% CI

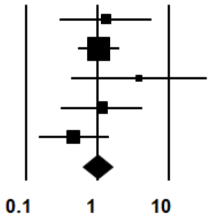

G AD - Haplogroup X

Study name	Odds ratio	Lower limit	Upper limit	p-Value
AD-2	1.347	0.536	3.385	0.527
AD-5	1.450	0.762	2.757	0.258
AD-9	0.934	0.058	15.021	0.962
AD-10	1.674	0.551	5.086	0.364
AD-13	0.256	0.010	6.340	0.405
Fixed	1.390	0.873	2.211	0.165

Odds ratio and 95% CI

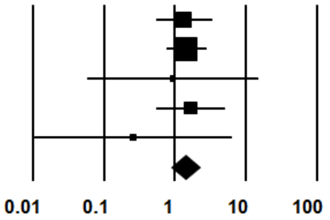

Supplementary Figure S1. Meta-analysis of previously reported AD and mtDNA haplogroup associations. A. Haplogroup T analysis. B. Haplogroup J analysis. C. Haplogroup K analysis. D. Haplogroup U analysis. E. Haplogroup V analysis. F. Haplogroup W analysis. G. Haplogroup X analysis.

A **Longevity - Haplogroup H**

Study name	Odds ratio	Lower limit	Upper limit	p-Value
Longevity 1-1	0.875	0.517	1.481	0.619
Longevity 1-2	1.217	0.730	2.028	0.452
Longevity 3	0.572	0.410	0.799	0.001
Longevity 9	1.668	0.920	3.024	0.092
Fixed	0.843	0.671	1.058	0.141

Odds ratio and 95% CI

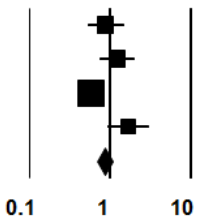

B **Longevity - Haplogroup I**

Study name	Odds ratio	Lower limit	Upper limit	p-Value
Longevity 1-1	0.760	0.125	4.635	0.766
Longevity 1-2	2.258	0.621	8.210	0.216
Longevity 3	1.438	0.559	3.697	0.451
Fixed	1.493	0.739	3.013	0.264

Odds ratio and 95% CI

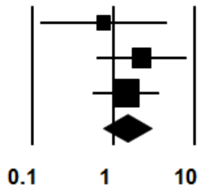

C **Longevity - Haplogroup K**

Study name	Odds ratio	Lower limit	Upper limit	p-Value
Longevity 1-1	1.155	0.361	3.693	0.808
Longevity 1-2	0.862	0.362	2.051	0.737
Longevity 3	1.280	0.672	2.438	0.452
Fixed	1.119	0.698	1.795	0.640

Odds ratio and 95% CI

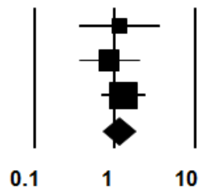

D **Longevity - Haplogroup T**

Study name	Odds ratio	Lower limit	Upper limit	p-Value
Longevity 1-1	0.907	0.405	2.033	0.813
Longevity 1-2	0.518	0.196	1.372	0.186
Longevity 3	0.883	0.420	1.857	0.743
Longevity 9	1.653	0.549	4.976	0.372
Fixed	0.882	0.570	1.366	0.575

Odds ratio and 95% CI

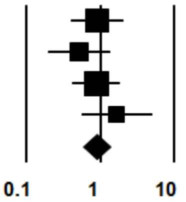

E **Longevity - Haplogroup U**

Study name	Odds ratio	Lower limit	Upper limit	p-Value
Longevity 1-1	0.860	0.397	1.863	0.703
Longevity 1-2	0.996	0.468	2.118	0.992
Longevity 3	1.542	1.056	2.250	0.025
Longevity 9	0.532	0.237	1.193	0.126
Fixed	1.163	0.871	1.552	0.307

Odds ratio and 95% CI

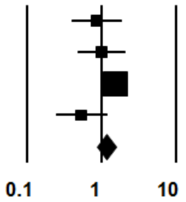

F **Longevity - Haplogroup V**

Study name	Odds ratio	Lower limit	Upper limit	p-Value
Longevity 1-1	1.148	0.071	18.577	0.923
Longevity 1-2	2.258	0.621	8.210	0.216
Longevity 3	0.434	0.143	1.316	0.140
Longevity 9	0.786	0.202	3.066	0.729
Fixed	0.865	0.433	1.729	0.681

Odds ratio and 95% CI

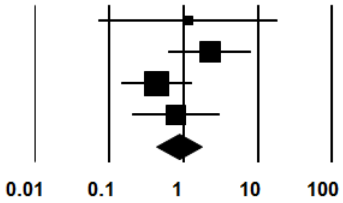

G **Longevity - Haplogroup X**

Study name	Odds ratio	Lower limit	Upper limit	p-Value
Longevity 1-1	1.151	0.227	5.824	0.865
Longevity 1-2	1.480	0.417	5.247	0.544
Longevity 3	1.276	0.400	4.068	0.680
Fixed	1.315	0.617	2.802	0.477

Odds ratio and 95% CI

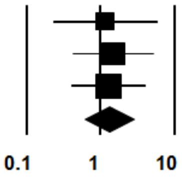

H Longevity – Asian - Haplogroup D

Study name	Odds ratio	Lower limit	Upper limit	p-Value
Longevity-7	1.267	0.765	2.099	0.358
Longevity-8	0.985	0.599	1.621	0.953
Longevity-13	1.989	0.979	4.042	0.057
Fixed	1.252	0.912	1.719	0.165

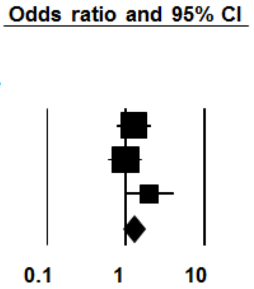

Supplementary Figure S2. Meta-analysis of previously reported Longevity mtDNA haplogroup associations. A. Haplogroup H analysis. B. Haplogroup I analysis. C. Haplogroup K analysis. D. Haplogroup T analysis. E. Haplogroup U analysis. F. Haplogroup V analysis. G. Haplogroup X analysis. H. Haplogroup D analysis.

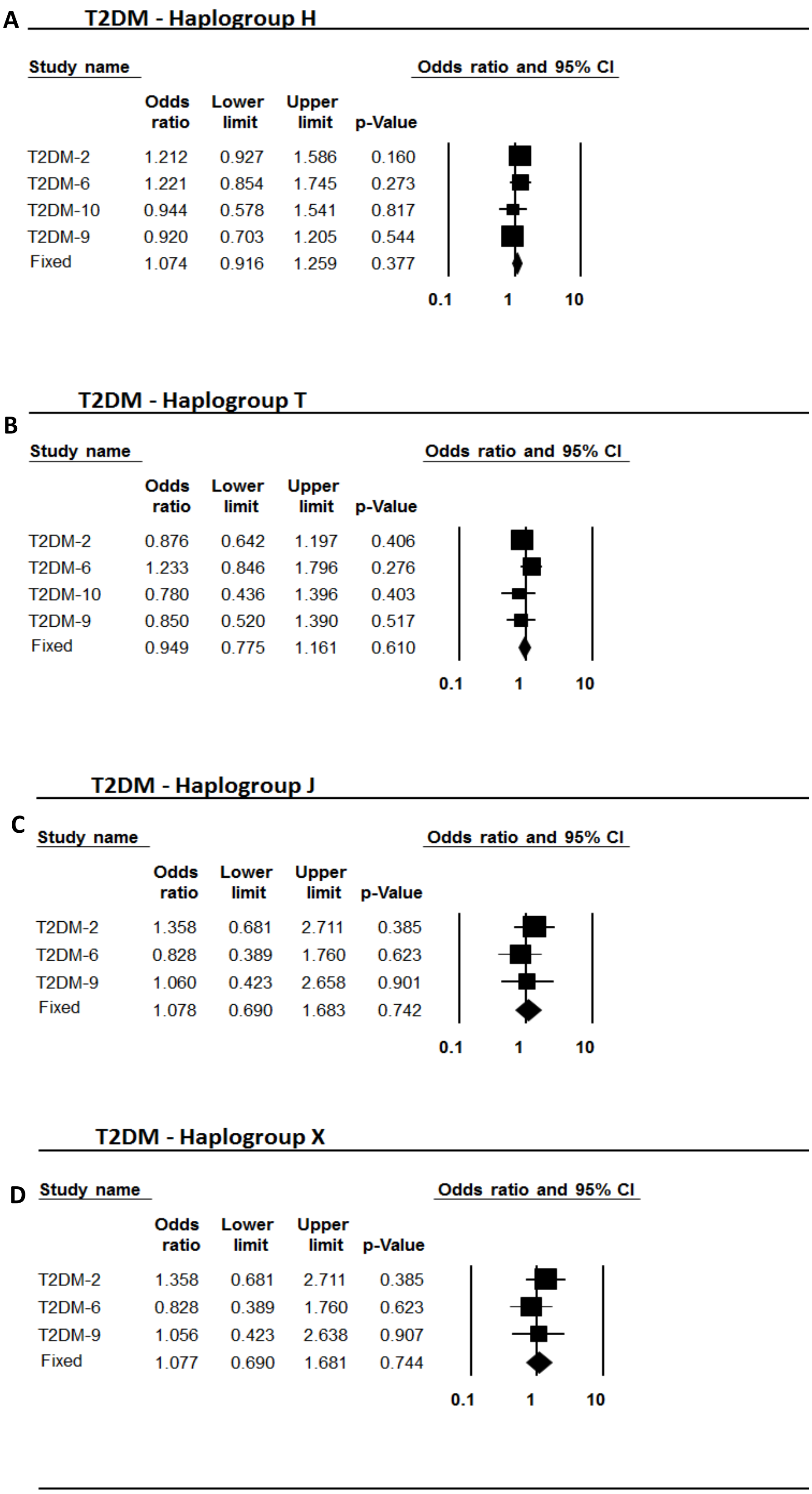

Supplementary Figure S3. Meta-analysis of previously reported T2DM in Caucasian population and mtDNA haplogroup associations. A. Haplogroup H analysis. B. Haplogroup T analysis. C. Haplogroup J analysis. D. Haplogroup X analysis.

A T2DM- Haplogroup D5

Study name	Odds ratio	Lower limit	Upper limit	p-Value
T2DM1	1.379	1.045	1.819	0.023
TSDM11	0.903	0.637	1.279	0.565
TSDM14	18.950	2.501	143.572	0.004
Fixed	1.208	0.974	1.499	0.086

Odds ratio and 95% CI

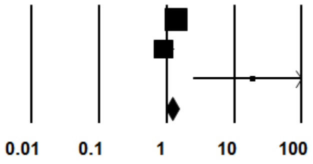

B T2DM - Haplogroup G

Study name	Odds ratio	Lower limit	Upper limit	p-Value
T2DM1	0.992	0.814	1.210	0.937
TSDM8	2.882	0.312	26.588	0.350
TSDM11	0.588	0.324	1.066	0.080
Fixed	0.949	0.787	1.145	0.585

Odds ratio and 95% CI

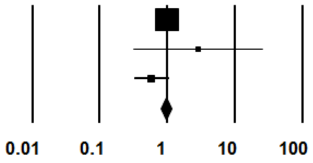

Supplementary Figure S4. Meta-analysis of previously reported T2DM in Asian population and mtDNA haplogroup associations. A. Haplogroup D5 analysis. B. Haplogroup G analysis.

A **Haplogroup I**

Study name	Odds ratio	Lower limit	Upper limit	p-Value
AD	1.280	0.484	3.387	0.619
TSDM	0.710	0.408	1.236	0.226
Sperm motility	1.487	0.297	7.444	0.629
Brest cancer	1.707	0.663	4.398	0.268
PC	1.083	0.464	2.528	0.854
AMD	1.183	0.625	2.239	0.605
Prostate Cancer	0.579	0.263	1.277	0.176
Schizo	0.498	0.045	5.531	0.570
PD	0.842	0.494	1.436	0.529
Fixed	0.924	0.714	1.197	0.551

Odds ratio and 95% CI

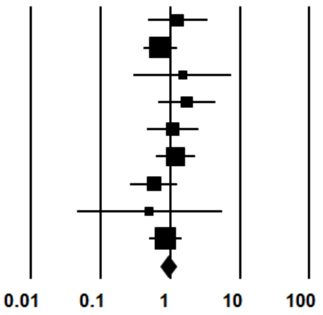

B **Haplogroup T**

Study name	Odds ratio	Lower limit	Upper limit	p-Value
AD	0.945	0.737	1.212	0.654
TSDM	1.055	0.849	1.311	0.630
Brest cancer	1.153	0.553	2.405	0.704
PC	0.926	0.656	1.307	0.661
AMD	1.099	0.743	1.625	0.637
Prostate Cancer	0.865	0.597	1.253	0.444
Schizo	1.213	0.512	2.874	0.661
PD	0.857	0.757	0.970	0.014
Fixed	0.922	0.844	1.007	0.070

Odds ratio and 95% CI

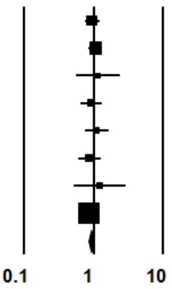

C **Haplogroup U**

Study name	Odds ratio	Lower limit	Upper limit	p-Value
AD	0.999	0.840	1.188	0.988
TSDM	1.055	0.849	1.311	0.630
Sperm motility	3.515	1.211	10.204	0.021
Brest cancer	1.153	0.553	2.405	0.704
CAD2	1.929	1.415	2.629	0.000
PC	0.926	0.656	1.307	0.661
AMD	1.099	0.743	1.625	0.637
Prostate Cancer	0.865	0.597	1.253	0.444
Schizo	1.213	0.512	2.874	0.661
PD	0.756	0.568	1.005	0.054
Fixed	1.050	0.952	1.158	0.331

Odds ratio and 95% CI

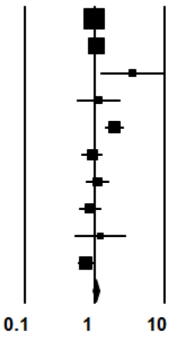

D **Haplogroup V**

Study name | Odds ratio and 95% CI

Study name	Odds ratio	Lower limit	Upper limit	p-Value
AD	0.994	0.689	1.433	0.974
TSDM	0.661	0.372	1.175	0.159
Sperm motility	0.986	0.391	2.487	0.976
Brest cancer	1.000	0.236	4.243	1.000
PC	1.005	0.591	1.710	0.984
AMD	1.562	0.894	2.730	0.118
Prostate Cancer	1.111	0.448	2.757	0.820
PD	1.159	0.685	1.962	0.582
Fixed	1.034	0.842	1.270	0.749

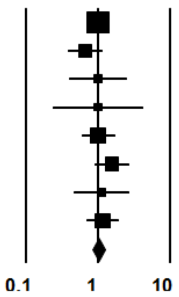

E **Haplogroup W**

Study name | Odds ratio and 95% CI

Study name	Odds ratio	Lower limit	Upper limit	p-Value
AD	1.044	0.649	1.679	0.858
TSDM	2.071	0.987	4.347	0.054
Sperm motility	0.986	0.179	5.435	0.987
Brest cancer	1.113	0.184	6.733	0.908
CAD2	0.880	0.417	1.856	0.736
PC	0.809	0.263	2.489	0.711
AMD	0.768	0.306	1.926	0.574
Prostate Cancer	0.695	0.262	1.839	0.463
Schizo	1.186	0.612	2.300	0.614
PD	0.784	0.366	1.678	0.531
Fixed	1.027	0.798	1.321	0.836

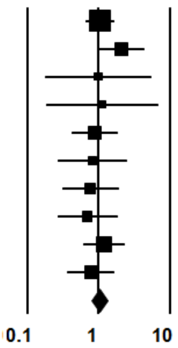

F **Haplogroup X**

Study name | Odds ratio and 95% CI

Study name	Odds ratio	Lower limit	Upper limit	p-Value
AD	1.386	0.872	2.201	0.167
TSDM	1.108	0.667	1.841	0.691
Sperm motility	1.154	0.295	4.515	0.837
Brest cancer	1.113	0.184	6.733	0.908
PC	1.039	0.407	2.652	0.937
AMD	0.671	0.159	2.828	0.586
Prostate Cancer	1.848	0.678	5.033	0.230
Schizo	0.498	0.045	5.531	0.570
PD	0.794	0.398	1.583	0.512
Fixed	1.141	0.876	1.487	0.327

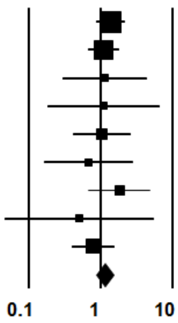

Supplementary Figure S5. MtDNA haplogroup association with grouped phenotypes. Forest plot for the analysis of the different phenotype. AD - Alzheimer's

disease, T2DM-type 2 diabetes mellitus, PC- prostate cancer, AMD - Age-related macular degeneration, Schizo – Schizophrenia, PD- Parkinson’s disease. A. Haplogroup I analysis. B. Haplogroup T analysis. C. Haplogroup U analysis. D. Haplogroup V analysis. E. Haplogroup W analysis. F. Haplogroup X analysis.

Supplementary Table S1. Search strategies

Search 1: PubMed, Scholar search through February 30, 2016 (" mitochondrial DNA association studies", "mitochondrial DNA haplogroup association studies", "mitochondrial DNA polymorphism")

Search 2 – per phenotype: PubMed, Scholar search through February 30, 2016

Alzheimer disease: "mitochondrial DNA association with Alzheimer disease", "mitochondrial DNA haplogroup association with Alzheimer disease"

Parkinson disease: "mitochondrial DNA association with Parkinson disease", "mitochondrial DNA haplogroup association with Parkinson disease"

Type 2 diabetes mellitus: "mitochondrial DNA association with T2DM", "mitochondrial DNA haplogroup association with T2DM ", " mitochondrial DNA association with Type 2 diabetes mellitus", "mitochondrial DNA haplogroup association with diabetes mellitus"

Schizophrenia: "mitochondrial DNA association with Schizophrenia", "mitochondrial DNA haplogroup association with Schizophrenia"

Longevity: " mitochondrial DNA association with Longevity", "mitochondrial DNA haplogroup association with Longevity"," mitochondrial DNA association with extreme longevity", "mitochondrial DNA haplogroup association with extreme longevity"

Age related macular degeneration (AMD): "mitochondrial DNA association with AMD", "mitochondrial DNA haplogroup association with AMD", "mitochondrial DNA association with Age related macular degeneration ", "mitochondrial DNA haplogroup association with Age related macular degeneration"

Sperm motility: "mitochondrial DNA association with sperm motility", "mitochondrial DNA haplogroup association with Sperm motility"

Cancer: "mitochondrial DNA association with cancer", "mitochondrial DNA haplogroup association with cancer"," mitochondrial DNA association with Breast cancer", "mitochondrial DNA haplogroup association with Breast cancer","mitochondrial DNA association with Prostate cancer motility", "mitochondrial DNA haplogroup association with Prostate cancer"

Supplementary Table S2. References for forest plot "study name". A. AD - Alzheimer's disease; B. PD- Parkinson's disease; C. T2DM-type 2 diabetes mellitus; D. Longevity; E. Breast Cancer; F. AMD - Age-related macular degeneration, MS - multiple sclerosis, MI - myocardial infarction, CAD - coronary artery disease and schizophrenia.

A.

Study name	Reference
AD 1	[1]
AD 2	[2]
AD 3	[3]
AD 4	[4]
AD 5	[5]
AD 6	[6]
AD 7	[7]
AD 8	[8]
AD 9	[9]
AD 10	[10]
AD 11	[11]
AD 12	[12]
AD 13	[13]
AD 14	[14]

B.

Study name	Reference
PD 1	[15]
PD 2	[16]
PD 3	[14]
PD 4	[17]
PD 5	[18]
PD 6	[19]
PD 7	[20]
PD 8	[21]
PD 9	[22]
PD 10	[23]
PD 11	[24]
PD 12	[25]
PD 3-1-1	[26]
PD 3-1-2	[26]

C.

Study name	Reference
T2DM 1	[27]
T2DM 2	[28]
T2DM 3	[29]
T2DM 4	[30]
T2DM 5	[31]
T2DM 6	[32]
T2DM 7	[33]
T2DM 8	[34]
T2DM 9	[35]
T2DM 10	[36]
T2DM 11	[37]
T2DM 12	[38]
T2DM 13	[39]
T2DM 14	[40]
T2DM 15	[41]

D.

Study name	Reference
Longevity 1	[42]
Longevity 2	[43]
Longevity 3	[44]
Longevity 4	[45]
Longevity 5	[46]
Longevity 6	[47]
Longevity 7	[48]
Longevity 8	[49]
Longevity 9	[50]
Longevity 10	[51]
Longevity 11	[52]
Longevity 12	[53]
Longevity 13	[54]
Longevity 14	[55]

E.

Study name	Reference
Breast cancer 1	56
Breast cancer 2	57
Breast cancer 3	58
Breast cancer 4	59
Breast cancer 5	60
Breast cancer 6	61
Breast cancer 7	62
Breast cancer 8	63
Breast cancer 9	64
Breast cancer 10	65
Breast cancer 11	66
Breast cancer 12	67
Breast cancer 13	68

F.

Study name	Reference
MS1	69
MS2	70
MS3	71
MS4	72
MI1	73
MI2	74
MI3	75
CAD1	76
CAD2	77
PC1	78
PC2	79
AMD1	80
AMD2	81
AMD3	82
AMD4	83
Schizophrenia1	84
Schizophrenia2	85
Sperm motility1	86
Sperm motility2	87
Sperm motility3	88
Sperm motility4	89
Prostate cancer1	90
Prostate cancer2	91
Prostate cancer3	92
Prostate cancer4	93
Prostate cancer5	94
Prostate cancer6	95

Supplementary Table S3. References for forest plot "study name". mtDNA haplogroup H association with grouped phenotypes. AD - Alzheimer's disease, T2DM-type 2 diabetes mellitus, PD- Parkinson's disease, PC - Pancreatic cancer, AMD - Age-related macular degeneration, CAD - coronary artery disease.

Study name	Reference
AD	2,5,10,13,14,32
T2DM	28,32,35
Breast cancer	56
Sperm motility	86
PC	78,79
AMD	83
CAD	77
Prostate cancer	91,93
PD	14,15,24

Supplementary Table S4. References for forest plot "study name". mtDNA haplogroup K association with grouped phenotypes. AD - Alzheimer's disease, T2DM-type 2 diabetes mellitus, PD- Parkinson's disease, PC - Pancreatic cancer, AMD - Age-related macular degeneration, CAD - coronary artery disease. Schizo – Schizophrenia.

Study name	Reference
AD	2,5,10,13,14,32
T2DM	28
Breast cancer	56
Sperm motility	86
PC	78,79
AMD	83
CAD	77
Schizo	84
Prostate cancer	91,93
PD	14,15,17,18,21-24,26

Supplementary Table S5. References for forest plot "study name". mtDNA haplogroup J association with grouped phenotypes. AD - Alzheimer's disease, T2DM- type 2 diabetes mellitus, PD- Parkinson's disease, PC - Pancreatic cancer, AMD - Age-related macular degeneration, CAD - coronary artery disease. Schizo – Schizophrenia.

Study name	Reference
AD	1,2,4,9,10,13,14
T2DM	28,32,35
Breast cancer	56
Sperm motility	86
PC	78,79
AMD	83
CAD	77
Schizo	84
Prostate cancer	91,93
PD	14-18,21-24,26

Supplementary Table S6. References for forest plot "study name". mtDNA haplogroup I association with grouped phenotypes. AD - Alzheimer's disease, T2DM- type 2 diabetes mellitus, PD- Parkinson's disease, PC - Pancreatic cancer, AMD - Age-related macular degeneration, CAD - coronary artery disease. Schizo – Schizophrenia.

Study name	Reference
AD	9,10,13
T2DM	32,36
Sperm motility	86
Breast cancer	56
PC	78
AMD	83
Prostate cancer	91-93
Schizo	84
PD	15,24

Supplementary Table S7. References for forest plot "study name". mtDNA haplogroup T association with grouped phenotypes. AD - Alzheimer's disease, T2DM-type 2 diabetes mellitus, PD- Parkinson's disease, PC - Pancreatic cancer, AMD - Age-related macular degeneration, CAD - coronary artery disease. Schizo – Schizophrenia.

Study name	Reference
AD	2,4,9,10,13,14,96
T2DM	28,32,35
Sperm motility	86
Breast cancer	56
PC	78,79
AMD	83
Prostate cancer	91,93
Schizo	84
PD	14,15,17,18,22-24,26

Supplementary Table S8. References for forest plot "study name". mtDNA haplogroup U association with grouped phenotypes. AD - Alzheimer's disease, T2DM-type 2 diabetes mellitus, PD- Parkinson's disease, PC - Pancreatic cancer, AMD - Age-related macular degeneration, CAD - coronary artery disease. Schizo – Schizophrenia.

Study name	Reference
AD	2,4,9,10,13,14,96
T2DM	28,32,35
Breast cancer	56
Sperm motility	86
PC	78,79
AMD	83
CAD	77
Prostate cancer	91,93
Schizo	84
PD	14,15,21,22,24

Supplementary Table S9. References for forest plot "study name". mtDNA haplogroup V association with grouped phenotypes. AD - Alzheimer's disease, T2DM-type 2 diabetes mellitus, PD- Parkinson's disease, PC - Pancreatic cancer, AMD - Age-related macular degeneration, CAD - coronary artery disease.

Study name	Reference
AD	2,5,10,13
T2DM	28
Breast cancer	56
Sperm motility	86
PC	78,79
AMD	83
Prostate cancer	91,93
PD	15,24

Supplementary Table S10. References for forest plot "study name". mtDNA haplogroup W association with grouped phenotypes. AD - Alzheimer's disease, T2DM-type 2 diabetes mellitus, PD- Parkinson's disease, PC - Pancreatic cancer, AMD - Age-related macular degeneration, CAD - coronary artery disease. Schizo – Schizophrenia.

Study name	Reference
AD	2,5,9,10,13
T2DM	28,32,36
Breast cancer	56
Sperm motility	86
PC	79
AMD	83
CAD	77
Prostate cancer	91,93
Schizo	84
PD	15,24

Supplementary Table S11. References for forest plot "study name". mtDNA haplogroup X association with grouped phenotypes. AD - Alzheimer's disease, T2DM-type 2 diabetes mellitus, PD- Parkinson's disease, PC - Pancreatic cancer, AMD - Age-related macular degeneration, CAD - coronary artery disease. Schizo – Schizophrenia.

Study name	Reference
AD	2,5,9,10,13
T2DM	28,32
Breast cancer	56
Sperm motility	86
PC	79
AMD	83
Prostate cancer	91,93
Schizo	84
PD	24,97

Supplementary Table S12. Example Data Extraction for Longevity (Longevity 3)

	Longevity			Non Longevity (controls)		
**Hap**	Haplogroup sample size	Others sample size	Total	Haplogroup sample size	Others sample size	Total
**H**	84	141	225	204	196	400
**I**	8	217	225	10	390	400
**J**	19	206	225	25	375	400
**K**	17	208	225	24	376	400
**M**	1	224	225	4	396	400
**T**	11	214	225	22	378	400
**U**	64	161	225	82	318	400
**V**	4	221	225	16	384	400
**W**	8	217	225	5	395	400
**X**	5	220	225	7	393	400

Supplementary Table S13. Longevity merged data and classification to haplogroups

	Haplogroup	Longevity			Non Longevity (Control)		
		Haplogroup sample size	Others sample size	Total	Haplogroup sample size	Others sample size	Total
**Longevity 1-1**	H	41	68	109	51	74	125
**Longevity 1-2**	H	44	59	103	57	93	150
**Longevity 3**	H	84	141	225	204	196	400
**Longevity 9**	H	37	28	65	61	77	138
**Longevity 1-1**	I	2	107	109	3	122	125
**Longevity 1-2**	I	6	97	103	4	146	150
**Longevity 3**	I	8	217	225	10	390	400
**Longevity 1-1**	J	17	92	109	6	119	125
**Longevity 1-2**	J	6	97	103	7	143	150
**Longevity 3**	J	19	206	225	25	375	400
**Longevity 9**	J	2	63	65	15	123	138
**Longevity 1-1**	K	6	103	109	6	119	125
**Longevity 1-2**	K	9	94	103	15	135	150
**Longevity 3**	K	17	208	225	24	376	400
**Longevity 1-1**	T	12	97	109	15	110	125
**Longevity 1-2**	T	6	97	103	16	134	150
**Longevity 3**	T	11	214	225	22	378	400
**Longevity 9**	T	6	59	65	8	130	138
**Longevity 1-1**	U	13	96	109	17	108	125
**Longevity 1-2**	U	13	90	103	19	131	150
**Longevity 3**	U	64	161	225	82	318	400
**Longevity 9**	U	9	56	65	32	106	138
**Longevity 1-1**	V	1	108	109	1	124	125
**Longevity 1-2**	V	6	97	103	4	146	150
**Longevity 3**	V	4	221	225	16	384	400
**Longevity 9**	V	3	62	65	8	130	138
**Longevity 1-1**	W	2	107	109	4	121	125

**Longevity 1-2**	W	1	102	103	6	144	150
**Longevity 3**	W	8	217	225	5	395	400
**Longevity 1-1**	X	3	106	109	3	122	125
**Longevity 1-2**	X	5	98	103	5	145	150
**Longevity 3**	X	5	220	225	7	393	400
**Longevity 4**	J2	8	38	46	3	54	57
**Longevity 3**	M	1	224	225	4	396	400
**Longevity 9**	O	1	64	65	14	124	138
**Longevity 4**	T2	1	45	46	4	53	57
**Longevity 4**	U5	34	12	46	48	9	57

Supporting References

1 Carrieri, G. *et al.* Mitochondrial DNA haplogroups and APOE4 allele are non-independent variables in sporadic Alzheimer's disease. *Hum Genet* **108**, 194-198. (2001).

2 Fachal, L. *et al.* No evidence of association between common European mitochondrial DNA variants in Alzheimer, Parkinson, and migraine in the Spanish population. *Am J Med Genet B Neuropsychiatr Genet* **168B**, 54-65, doi:10.1002/ajmg.b.32276 (2015).

3 Bi, R. *et al.* Mitochondrial DNA haplogroup B5 confers genetic susceptibility to Alzheimer's disease in Han Chinese. *Neurobiol Aging* **36**, 1604 e1607-1616, doi:10.1016/j.neurobiolaging.2014.10.009 (2015).

4 Fesahat, F., Houshmand, M., Panahi, M. S., Gharagozli, K. & Mirzajani, F. Do haplogroups H and U act to increase the penetrance of Alzheimer's disease? *Cell Mol Neurobiol* **27**, 329-334, doi:10.1007/s10571-006-9126-9 (2007).

5 Santoro, A. *et al.* Evidence for sub-haplogroup h5 of mitochondrial DNA as a risk factor for late onset Alzheimer's disease. *PLoS One* **5**, e12037, doi:10.1371/journal.pone.0012037 (2010).

6 Chagnon, P. *et al.* Phylogenetic analysis of the mitochondrial genome indicates significant differences between patients with Alzheimer disease and controls in a French-Canadian founder population. *Am J Med Genet* **85**, 20-30 (1999).

7 Chinnery, P. F. *et al.* Point mutations of the mtDNA control region in normal and neurodegenerative human brains. *Am J Hum Genet* **68**, 529-532, doi:10.1086/318204 (2001).

8 Coppede, F. *et al.* A Ser326Cys polymorphism in the DNA repair gene hOGG1 is not associated with sporadic Alzheimer's disease. *Neurosci Lett* **414**, 282-285, doi:10.1016/j.neulet.2006.12.035 (2007).

9 Elson, J. L. *et al.* Does the mitochondrial genome play a role in the etiology of Alzheimer's disease? *Hum Genet* **119**, 241-254, doi:10.1007/s00439-005-0123-8 (2006).

10 Mancuso, M. *et al.* Lack of association between mtDNA haplogroups and Alzheimer's disease in Tuscany. *Neurol Sci* **28**, 142-147, doi:10.1007/s10072-007-0807-z (2007).

11 van der Walt, J. M. *et al.* Analysis of European mitochondrial haplogroups with Alzheimer disease risk. *Neurosci Lett* **365**, 28-32, doi:10.1016/j.neulet.2004.04.051 (2004).

12 Lakatos, A. *et al.* Association between mitochondrial DNA variations and Alzheimer's disease in the ADNI cohort. *Neurobiol Aging* **31**, 1355-1363, doi:10.1016/j.neurobiolaging.2010.04.031 (2010).

13 Kruger, J., Hinttala, R., Majamaa, K. & Remes, A. M. Mitochondrial DNA haplogroups in early-onset Alzheimer's disease and frontotemporal lobar degeneration. *Mol Neurodegener* **5**, 8, doi:10.1186/1750-1326-5-8 (2010).

14 Pyle, A. *et al.* Mitochondrial DNA haplogroup cluster UKJT reduces the risk of PD. *Ann Neurol* **57**, 564-567 (2005).

15 van der Walt, J. M. *et al.* Mitochondrial polymorphisms significantly reduce the risk of Parkinson disease. *Am J Hum Genet* **72**, 804-811. (2003).

16 Ross, O. A. *et al.* mt4216C variant in linkage with the mtDNA TJ cluster may confer a susceptibility to mitochondrial dysfunction resulting in an increased risk of Parkinson's disease in the Irish. *Exp Gerontol* **38**, 397-405 (2003).

17 Huerta, C. *et al.* Mitochondrial DNA polymorphisms and risk of Parkinson's disease in Spanish population. *J Neurol Sci* **236**, 49-54 (2005).

18 Ghezzi, D. *et al.* Mitochondrial DNA haplogroup K is associated with a lower risk of Parkinson's disease in Italians. *Eur J Hum Genet* **13**, 748-752 (2005).

19 Autere, J. *et al.* Mitochondrial DNA polymorphisms as risk factors for Parkinson's disease and Parkinson's disease dementia. *Hum Genet* **115**, 29-35, doi:10.1007/s00439-004-1123-9 (2004).

20 Gusdon, A. M. *et al.* Association of the mt-ND2 5178A/C polymorphism with Parkinson's disease. *Neurosci Lett* **587**, 98-101, doi:10.1016/j.neulet.2014.12.005 (2015).

21 Mehta, P. *et al.* Mitochondrial DNA haplogroups J and K are not protective for Parkinson's disease in the Australian community. *Mov Disord* **24**, 290-292, doi:10.1002/mds.22389 (2009).

22 Gaweda-Walerych, K. *et al.* Mitochondrial DNA haplogroups and subhaplogroups are associated with Parkinson's disease risk in a Polish PD cohort. *J Neural Transm (Vienna)* **115**, 1521-1526, doi:10.1007/s00702-008-0121-9 (2008).

23 Simon, D. K. *et al.* Mitochondrial DNA mutations in complex I and tRNA genes in Parkinson's disease. *Neurology* **54**, 703-709 (2000).

24 Latsoudis, H., Spanaki, C., Chlouverakis, G. & Plaitakis, A. Mitochondrial DNA polymorphisms and haplogroups in Parkinson's disease and control individuals with a similar genetic background. *J Hum Genet* **53**, 349-356, doi:10.1007/s10038-008-0259-1 (2008).

25 Simon-Sanchez, J. *et al.* Genome-wide association study reveals genetic risk underlying Parkinson's disease. *Nat Genet* **41**, 1308-1312, doi:10.1038/ng.487 (2009).

26 Hudson, G. *et al.* Two-stage association study and meta-analysis of mitochondrial DNA variants in Parkinson disease. *Neurology* **80**, 2042-2048, doi:10.1212/WNL.0b013e318294b434 (2013).

27 Fuku, N. *et al.* Mitochondrial haplogroup N9a confers resistance against type 2 diabetes in Asians. *Am J Hum Genet* **80**, 407-415, doi:10.1086/512202 (2007).

28 Chinnery, P. F. *et al.* Mitochondrial DNA haplogroups and type 2 diabetes: a study of 897 cases and 1010 controls. *J Med Genet* **44**, e80, doi:10.1136/jmg.2007.048876 (2007).

29 Park, K. S. *et al.* A mitochondrial DNA variant at position 16189 is associated with type 2 diabetes mellitus in Asians. *Diabetologia* **51**, 602-608, doi:10.1007/s00125-008-0933-z (2008).

30 Poulton, J. *et al.* Type 2 diabetes is associated with a common mitochondrial variant: evidence from a population-based case-control study. *Hum Mol Genet* **11**, 1581-1583 (2002).

31 Tang, D. L., Zhou, X., Li, X., Zhao, L. & Liu, F. Variation of mitochondrial gene and the association with type 2 diabetes mellitus in a Chinese population. *Diabetes Res Clin Pract* **73**, 77-82, doi:10.1016/j.diabres.2005.12.001 (2006).

32 Achilli, A. *et al.* Mitochondrial DNA backgrounds might modulate diabetes complications rather than T2DM as a whole. *PLoS One* **6**, e21029 (2011).

33 Martikainen, M. H., Ronnemaa, T. & Majamaa, K. Association of mitochondrial DNA haplogroups and vascular complications of diabetes mellitus: A population-based study. *Diab Vasc Dis Res* **12**, 302-304, doi:10.1177/1479164115579007 (2015).

34 Liao, W. Q. *et al.* Novel mutations of mitochondrial DNA associated with type 2 diabetes in Chinese Han population. *Tohoku J Exp Med* **215**, 377-384 (2008).

35 Mohlke, K. L. *et al.* Mitochondrial polymorphisms and susceptibility to type 2 diabetes-related traits in Finns. *Hum Genet* **118**, 245-254, doi:10.1007/s00439-005-0046-4 (2005).

36 Feder, J. *et al.* Parental diabetes status reveals association of mitochondrial DNA haplogroup J1 with type 2 diabetes. *BMC Med Genet* **10**, 60 (2009).

37 Liou, C. W. *et al.* Mitochondrial DNA coding and control region variants as genetic risk factors for type 2 diabetes. *Diabetes* **61**, 2642-2651, doi:10.2337/db11-1369 (2012).
38 Wang, S. *et al.* Mitochondrial DNA mutations in diabetes mellitus patients in Chinese Han population. *Gene* **531**, 472-475, doi:10.1016/j.gene.2013.09.019 (2013).
39 Yang, X., Zhang, Y., Ma, Y., Zhao, Q. & Lyu, J. [Effect of mitochondrial DNA 5178 C/A polymorphism on risks for type 2 diabetes mellitus and its complications]. *Zhonghua Yi Xue Yi Chuan Xue Za Zhi* **32**, 855-860, doi:10.3760/cma.j.issn.1003-9406.2015.06.023 (2015).
40 Niu, Q. *et al.* Effects of mitochondrial haplogroup N9a on type 2 diabetes mellitus and its associated complications. *Exp Ther Med* **10**, 1918-1924, doi:10.3892/etm.2015.2751 (2015).
41 Liou, C. W. *et al.* A common mitochondrial DNA variant and increased body mass index as associated factors for development of type 2 diabetes: Additive effects of genetic and environmental factors. *J Clin Endocrinol Metab* **92**, 235-239, doi:10.1210/jc.2006-0653 (2007).
42 De Benedictis, G. *et al.* Mitochondrial DNA inherited variants are associated with successful aging and longevity in humans. *Faseb J* **13**, 1532-1536. (1999).
43 Dato, S. *et al.* Association of the mitochondrial DNA haplogroup J with longevity is population specific. *Eur J Hum Genet* **12**, 1080-1082 (2004).
44 Niemi, A. K. *et al.* Mitochondrial DNA polymorphisms associated with longevity in a Finnish population. *Hum Genet* **112**, 29-33. (2003).
45 Niemi, A. K. *et al.* A combination of three common inherited mitochondrial DNA polymorphisms promotes longevity in Finnish and Japanese subjects. *Eur J Hum Genet* **13**, 166-170, doi:10.1038/sj.ejhg.5201308 (2005).
46 Yao, Y. G., Kong, Q. P. & Zhang, Y. P. Mitochondrial DNA 5178A polymorphism and longevity. *Hum Genet* **111**, 462-463, doi:10.1007/s00439-002-0826-z (2002).
47 Rose, G. *et al.* Paradoxes in longevity: sequence analysis of mtDNA haplogroup J in centenarians. *Eur J Hum Genet* **9**, 701-707. (2001).
48 Feng, J. *et al.* Association of mtDNA haplogroup F with healthy longevity in the female Chuang population, China. *Exp Gerontol* **46**, 987-993, doi:10.1016/j.exger.2011.09.001 (2011).
49 Alexe, G. *et al.* Enrichment of longevity phenotype in mtDNA haplogroups D4b2b, D4a, and D5 in the Japanese population. *Hum Genet* **121**, 347-356, doi:10.1007/s00439-007-0330-6 (2007).
50 Pinos, T. *et al.* Are mitochondrial haplogroups associated with extreme longevity? A study on a Spanish cohort. *Age (Dordr)* **34**, 227-233, doi:10.1007/s11357-011-9209-5 (2012).
51 Takasaki, S. Mitochondrial haplogroups associated with Japanese centenarians, Alzheimer's patients, Parkinson's patients, type 2 diabetic patients and healthy non-obese young males. *J Genet Genomics* **36**, 425-434 (2009).
52 Cai, X. Y. *et al.* Association of mitochondrial DNA haplogroups with exceptional longevity in a Chinese population. *PLoS One* **4**, e6423 (2009).
53 Bilal, E. *et al.* Mitochondrial DNA haplogroup D4a is a marker for extreme longevity in Japan. *PLoS One* **3**, e2421, doi:10.1371/journal.pone.0002421 (2008).
54 Tanaka, M., Gong, J. S., Zhang, J., Yoneda, M. & Yagi, K. Mitochondrial genotype associated with longevity. *Lancet* **351**, 185-186. (1998).
55 Tanaka, M. *et al.* Mitochondrial genotype associated with longevity and its inhibitory effect on mutagenesis. *Mech Ageing Dev* **116**, 65-76 (2000).

56 Bai, R. K., Leal, S. M., Covarrubias, D., Liu, A. & Wong, L. J. Mitochondrial genetic background modifies breast cancer risk. *Cancer Res* **67**, 4687-4694, doi:10.1158/0008-5472.CAN-06-3554 (2007).
57 Fang, H. *et al.* Cancer type-specific modulation of mitochondrial haplogroups in breast, colorectal and thyroid cancer. *BMC Cancer* **10**, 421, doi:10.1186/1471-2407-10-421 (2010).
58 Czarnecka, A. M. *et al.* Mitochondrial NADH-dehydrogenase subunit 3 (ND3) polymorphism (A10398G) and sporadic breast cancer in Poland. *Breast Cancer Res Treat* **121**, 511-518, doi:10.1007/s10549-009-0358-5 (2010).
59 Setiawan, V. W. *et al.* Mitochondrial DNA G10398A variant is not associated with breast cancer in African-American women. *Cancer Genet Cytogenet* **181**, 16-19 (2008).
60 Francis, A. *et al.* A mitochondrial DNA variant 10398G > A in breast cancer among South Indians: An original study with meta-analysis. *Mitochondrion* **13**, 559-565, doi:10.1016/j.mito.2013.08.004 (2013).
61 Covarrubias, D., Bai, R. K., Wong, L. J. & Leal, S. M. Mitochondrial DNA variant interactions modify breast cancer risk. *J Hum Genet* **53**, 924-928, doi:10.1007/s10038-008-0331-x (2008).
62 Canter, J. A., Kallianpur, A. R., Parl, F. F. & Millikan, R. C. Mitochondrial DNA G10398A polymorphism and invasive breast cancer in African-American women. *Cancer Res* **65**, 8028-8033 (2005).
63 Mosquera-Miguel, A. *et al.* Is mitochondrial DNA variation associated with sporadic breast cancer risk? *Cancer Res* **68**, 623-625; author reply 624, doi:10.1158/0008-5472.CAN-07-2385 (2008).
64 Jiang, H. *et al.* Peripheral blood mitochondrial DNA content, A10398G polymorphism, and risk of breast cancer in a Han Chinese population. *Cancer Sci* **105**, 639-645, doi:10.1111/cas.12412 (2014).
65 Mims, M. P. *et al.* Mitochondrial DNA G10398A polymorphism and invasive breast cancer in African-American women. *Cancer Res* **66**, 1880; author reply 1880-1881, doi:10.1158/0008-5472.CAN-05-3774 (2006).
66 Darvishi, K., Sharma, S., Bhat, A. K., Rai, E. & Bamezai, R. N. Mitochondrial DNA G10398A polymorphism imparts maternal Haplogroup N a risk for breast and esophageal cancer. *Cancer Lett* **249**, 249-255 (2007).
67 Pezzotti, A. *et al.* The mitochondrial A10398G polymorphism, interaction with alcohol consumption, and breast cancer risk. *PLoS One* **4**, e5356 (2009).
68 Tengku Baharudin, N., Jaafar, H. & Zainuddin, Z. Association of mitochondrial DNA 10398 polymorphism in invasive breast cancer in malay population of peninsular malaysia. *Malays J Med Sci* **19**, 36-42 (2012).
69 Houshmand, M. *et al.* Population screening for association of mitochondrial haplogroups BM, J, K and M with multiple sclerosis: interrelation between haplogroup J and MS in Persian patients. *Mult Scler J* **11**, 728-730, doi:10.1191/1352458505ms1228sr (2005).
70 Tranah, G. J. *et al.* Mitochondrial DNA sequence variation in multiple sclerosis. *Neurology* **85**, 325-330, doi:10.1212/WNL.0000000000001744 (2015).
71 Hassani-Kumleh, H. *et al.* Mitochondrial D-loop variation in Persian Multiple Sclerosis patients: K and A haplogroups as a risk factor!! *Cell Mol Neurobiol* **26**, 119-125, doi:10.1007/s10571-006-9026-z (2006).
72 Ghabaee, M. *et al.* Mitochondrial Mutation in Iranian Patients with Multiple Sclerosis, Correlation Between Haplogroups H, A and Clinical Manifestations. *Cell Mol Neurobiol* **29**, 341-346, doi:10.1007/s10571-008-9325-7 (2009).

73 Oguri, M. *et al.* Mitochondrial haplogroup N9b is protective against myocardial infarction in Japanese males. *J Am Coll Cardiol* **49**, 198a-198a (2007).
74 Takagi, K. *et al.* Association of a 5178C -> A (Leu237Met) polymorphism in the mitochondrial DNA with a low prevalence of myocardial infarction in Japanese individuals. *Atherosclerosis* **175**, 281-286, doi:10.1016/j.atherosclerosis.2004.03.008 (2004).
75 Abu-Amero, K. K. *et al.* The Mitochondrial DNA Variant 16189T > C Is Associated with Coronary Artery Disease and Myocardial Infarction in Saudi Arabs. *Genet Test Mol Bioma* **14**, 43-47, doi:10.1089/gtmb.2009.0095 (2010).
76 Mueller, E. E. *et al.* The Mitochondrial T16189C Polymorphism Is Associated with Coronary Artery Disease in Middle European Populations. *PLoS One* **6**, doi:ARTN e16455 10.1371/journal.pone.0016455 (2011).
77 Kofler, B. *et al.* Mitochondrial DNA haplogroup T is associated with coronary artery disease and diabetic retinopathy: a case control study. *BMC Med Genet* **10**, 35, doi:10.1186/1471-2350-10-35 (2009).
78 Lam, E. T. *et al.* Mitochondrial DNA sequence variation and risk of pancreatic cancer. *Cancer Res* **72**, 686-695, doi:10.1158/0008-5472.CAN-11-1682 (2012).
79 Wang, L. *et al.* Mitochondrial genetic polymorphisms and pancreatic cancer risk. *Cancer Epidemiol Biomarkers Prev* **16**, 1455-1459, doi:10.1158/1055-9965.EPI-07-0119 (2007).
80 Mueller, E. E. *et al.* Mitochondrial haplogroups and control region polymorphisms in age-related macular degeneration: a case-control study. *PLoS One* **7**, e30874, doi:10.1371/journal.pone.0030874 (2012).
81 Udar, N. *et al.* Mitochondrial DNA haplogroups associated with age-related macular degeneration. *Invest Ophthalmol Vis Sci* **50**, 2966-2974, doi:10.1167/iovs.08-2646 (2009).
82 Kenney, M. C. *et al.* Mitochondrial DNA haplogroups confer differences in risk for age-related macular degeneration: a case control study. *BMC Med Genet* **14**, 4, doi:10.1186/1471-2350-14-4 (2013).
83 Jones, M. M. *et al.* Mitochondrial DNA haplogroups and age-related maculopathy. *Arch Ophthalmol* **125**, 1235-1240 (2007).
84 Amar, S. *et al.* Mitochondrial DNA HV lineage increases the susceptibility to schizophrenia among Israeli Arabs. *Schizophr Res* **94**, 354-358 (2007).
85 Ueno, H. *et al.* Analysis of mitochondrial DNA variants in Japanese patients with schizophrenia. *Mitochondrion* **9**, 385-393, doi:10.1016/j.mito.2009.06.003 (2009).
86 Ruiz-Pesini, E. *et al.* Human mtDNA haplogroups associated with high or reduced spermatozoa motility. *Am J Hum Genet* **67**, 682-696, doi:10.1086/303040 (2000).
87 Montiel-Sosa, F. *et al.* Differences of sperm motility in mitochondrial DNA haplogroup U sublineages. *Gene* **368**, 21-27 (2006).
88 Ruiz-Pesini, E., Mishmar, D., Brandon, M., Procaccio, V. & Wallace, D. C. Effects of purifying and adaptive selection on regional variation in human mtDNA. *Science* **303**, 223-226, doi:DOI 10.1126/science.1088434 (2004).
89 Pereira, L. *et al.* No evidence for an mtDNA role in sperm motility: Data from complete sequencing of asthenozoospermic males. *Mol Biol Evol* **24**, 868-874, doi:10.1093/molbev/msm004 (2007).
90 Kim, W. *et al.* Mitochondrial DNA Haplogroup Analysis Reveals no Association between the Common Genetic Lineages and Prostate Cancer in the Korean Population. *PLoS One* **3**, doi:ARTN e2211 10.1371/journal.pone.0002211 (2008).

91 Mueller, E. E. *et al.* Mitochondrial haplogroups and control region polymorphisms are not associated with prostate cancer in Middle European Caucasians. *PLoS One* **4**, e6370 (2009).
92 Fachal, L. *et al.* No association between typical European mitochondrial variation and prostate cancer risk in a Spanish cohort. *Journal of Human Genetics* **59**, 411-414, doi:10.1038/jhg.2014.46 (2014).
93 Booker, L. M. *et al.* North American white mitochondrial haplogroups in prostate and renal cancer. *J Urol* **175**, 468-472; discussion 472-463, doi:10.1016/S0022-5347(05)00163-1 (2006).
94 Ray, A. M. *et al.* Sequence variation in the mitochondrial gene cytochrome c oxidase subunit I and prostate cancer in African American men. *Prostate* **69**, 956-960, doi:10.1002/pros.20943 (2009).
95 McCrow, J. P. *et al.* Spectrum of mitochondrial genomic variation and associated clinical presentation of prostate cancer in South African men. *Prostate* **76**, 349-358, doi:10.1002/pros.23126 (2016).
96 Fesahat, F., Houshmand, M., Panahi, M. S. S., Gharagozli, K. & Mirzajani, F. Do haplogroups H and U act to increase the penetrance of Alzheimer's disease? *Cell Mol Neurobiol* **27**, 329-334, doi:10.1007/s10571-006-9126-9 (2007).
97 van der Walt, J. M. *et al.* Mitochondrial polymorphisms significantly reduce the risk of Parkinson disease. *Am J Hum Genet* **72**, 804-811 (2003).
